# Electrically Stimulated Fire Ants (*Solenopsis invicta*, Hymenoptera: Formicidae) Release Chemical Cues That Attract Parasitic *Pseudacteon* Decapitating Flies (Diptera: Phoridae)

**DOI:** 10.1007/s10886-026-01710-w

**Published:** 2026-05-13

**Authors:** Robert K. Vander Meer, Sanford D. Porter, Yasmin J. Cardoza

**Affiliations:** 1 USDA-ARS, CMAVE, 1600 SW 23rd Drive, Gainesville, FL 32608 USA; 2 Retired USDA, Gainesville, FL USA; 3 Raleigh, NC USA

**Keywords:** Biocontrol, Semiochemicals, Parasitic behavior

## Abstract

We previously determined that electrically stimulated fire ants release exocrine gland products, e.g., venom, recruitment and alarm pheromones. We hypothesized that exocrine gland emissions from electrically stimulated fire ant workers might attract phorid fly parasitoids of fire ants. Bioassays demonstrated that electrically stimulated worker ants did attract phorid fly parasitoids in the field. Phorid fly (*Pseudacteon tricuspis*) mass rearing boxes were then used for lab bioassays to determine the glandular source and active components from electrically stimulated fire ants that elicited phorid fly attraction. The crude extract of fire ant poison sacs showed a significant increase in attacking phorid flies at the 10 min evaluation time, whereas purified venom alkaloids had no effect on phorid flies. Dufour’s glands (the source of recruitment pheromones) elicited no fly activation/attraction. However, mandibular gland extracts, the source of alarm pheromones, were found to activate and attract phorid flies. Moreover, a previously identified pyrazine alarm pheromone from mandibular glands was found to be the active component. This was confirmed by specific alarm pheromone bioassays showing the expected ephemeral characteristic of the attraction and by directly using synthetic fire ant alarm pheromone. Use of the fire ant alarm pheromone may aid in detecting the presence of phorid flies in the field and in increasing attack/parasitism rates during mass production.

## Introduction

More than 40 species of *Pseudacteon* decapitating flies are known to parasitize *Solenopsis saevissima* and *Solenopsis geminata* species complexes in the Americas (Plowes 2009, Patrock 2009). Once female flies locate their hosts, they hover above the ants until they are oriented properly above their target. Then in an instant they inject a single egg into the thorax of the ant (Porter 1998). This egg hatches within days and the maggot migrates into the ant head where it grows for 2–3 weeks before decapitating its host and pupating in the empty head capsule.

Several studies have examined chemicals that phorid flies use to locate their ant hosts. A parasitoid of the giant ant *Paraponera clavata* is attracted by mandibular gland alarm pheromones released by fighting or wounded ants (Feener et al. 1996). The leaf cutter ant parasitoid, *Neodohrniphora elongata* apparently relies primarily on visual cues, but trail pheromones increased fly motivation (Gazal et al. 2009). A *Pseudacteon* fly that parasitizes *Lasius* ants in Europe is attracted to its host by formic acid released by the venom gland of its host (Maschwitz et al. 2008). In contrast, *Pseudacteon brevicauda* is attracted to several components of the mandibular gland of its host ant, *Myrmica rubra* (Witte et al. 2010). The fire ant decapitating fly *Pseudacteon tricuspis* is known to be attracted to disturbed fire ants in the field that are presumably releasing alarm pheromones (Morrison and King 2004). Chen and Fadamiro (2007) confirmed that *P. tricuspis* was attracted to fire ant chemicals. They implicated the thorax as the source of the attractant compounds and ruled out the recruitment pheromone as a host location cue. However, they tested the weakly active but commercially available (*E*,* E*)-$\:\propto\:$-farnesene rather than the highly active (*Z*,* E*)-$\:\propto\:$-farnesene (Alonso and Vander Meer 1997). Sharma and Fadamiro (2013) showed that a combination of fire ant venom alkaloids and alarm pheromone elicited greater phorid fly (*Pseudacteon tricuspis*, *P. obtusus*, and *P. curvatus*) attraction than either alone. Ngumbi and Fadamiro (2015), showed that synthetic alarm pheromone and an isomer were attractive to four *Pseudacteon* species: *P. cultellatus*, *P. curvatus*, *P. obtusus*, and *P. tricuspis*.

Previous research demonstrated that electrically stimulated fire ant workers release venom gland contents, as well as recruitment and alarm pheromones (Vander Meer et al. 2002). The first objective of this study was to determine if phorid flies respond to volatiles released by electrically stimulated workers under field conditions. We then used phorid fly host seeking behavior in laboratory rearing attack boxes as a tool to ascertain the source(s) and nature of the semiochemicals involved in phorid fly attraction to their fire ant hosts.

## Methods and Materials

### Field Evaluation of Phorid Fly Attraction to Electrically Stimulated Fire Ant Workers

**Fig. 1:**
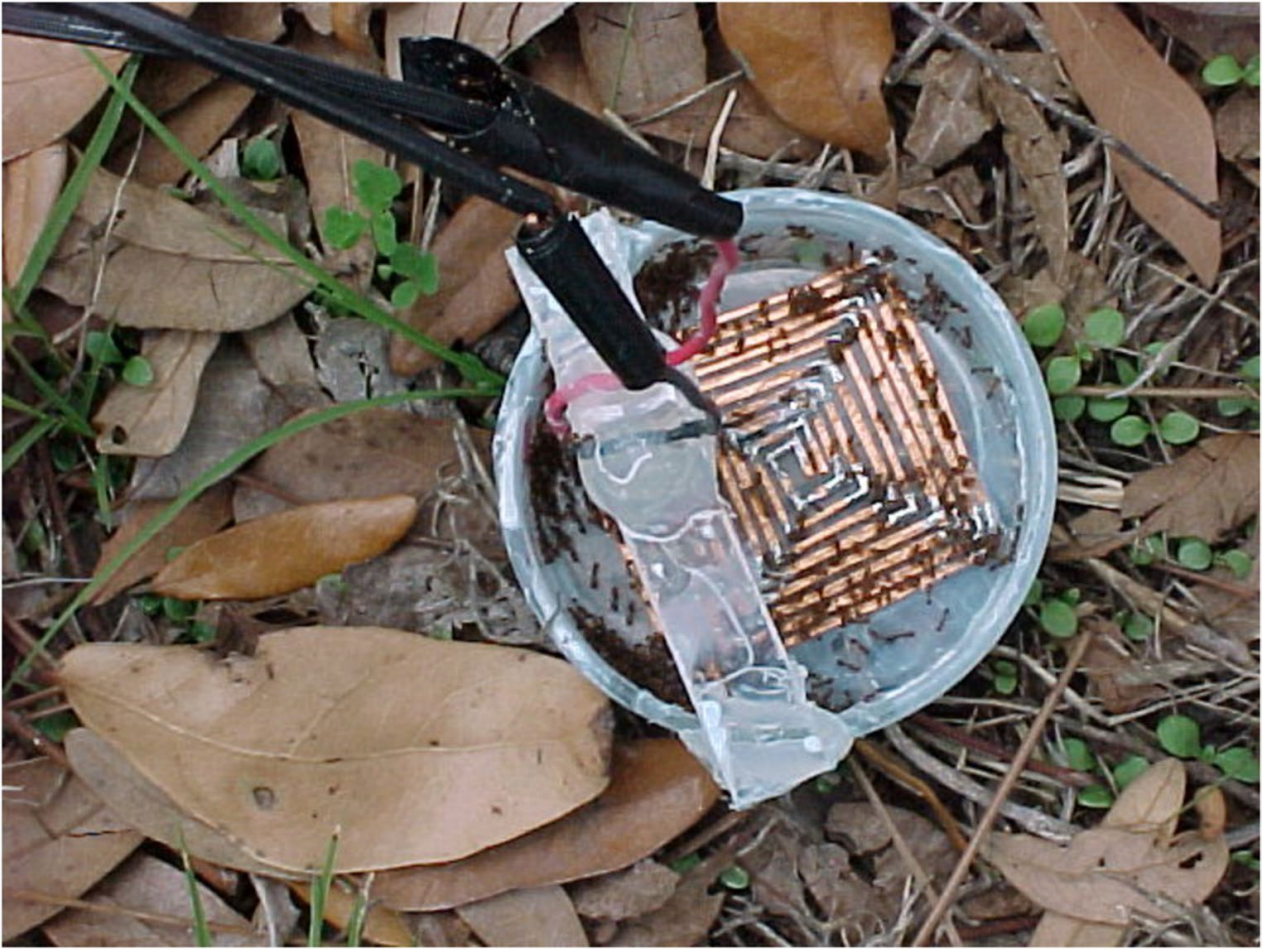
Treatment Petri dish fitted with two copper tape electrical grids. The grids were linked to an electrical source consisting of a voltage inverter and battery resulting in 110 V AC output adjusted to 30 V with a voltage regulator. Control Petri dishes did not contain the electrical grid

A field site with an established population of *P. tricuspis* flies (Hogtown Creek, Gainesville, FL; 29.6396° N, -82,3945° W) was selected for evaluation of the ability of electrically stimulated fire ants to attract phorid flies in their natural habitat. Petri Dishes (6 cm dia.) were fitted with two independent electrical grids (ca. 4 cm square) composed of 2 mm wide copper tape. The two grid patterns followed each other with spacing such that a walking fire ant worker would have a high probability of touching the two grids simultaneously, thus closing an electrical circuit. A standard electrical cord was soldered to the ends of the two independent copper tape grids in the Petri dish and braced in place with a small piece of Plexiglas (Fig. 1). Control Petri dishes were the same size as the treatment dishes, but without the electrical grid. The inner and outer sides of the treatment and control Petri dishes were coated with Fluon™ to prevent fire ant movement into or out of the Petri dishes. Electrical power was supplied using a voltage inverter to convert 12 V DC from a car battery into 110 V AC output adjusted to 30 V with a Variac voltage regulator (ISE, Cleveland, OH). A long extension cord carried the electrical power to the treatment Petri dishes.

The treatment (Fig. 1) and control Petri dishes were placed on the ground (3–4 m apart) at naturally shady spots in a clearing without regard to the location of existing fire ant colonies. Fire ant workers (100–200) from laboratory reared colonies were gently placed into the control and treatment Petri dishes. Counts of phorid fly activity at control and treatment Petri dishes were recorded (time 0), then the workers in the treatment Petri dish were electrically stimulated by turning the power on for 30 s, then off for 30 s for a total of three minutes. Observers experienced at detecting phorid flies in the field were at the treatment and control locations. The number of phorid flies at the Petri dishes was recorded at 3 and 8 min after the end of the electrical stimulation sequence. The experiment was replicated eight times at different locations within the field site.

### The Phorid Fly Rearing Box Bioassay

#### Fire Ants

All fire ants used in these experiments were from mature, queenright, monogyne *Solenopsis invicta* Buren colonies excavated from locations in and around Gainesville, FL. The colonies were separated from the dirt and set up in the laboratory at least one month prior to the experiments described in this paper. The colonies were maintained on a diet of crickets, water and 10% sucrose, and kept in plastic trays (83 × 53 × 13 cm) whose inner sides were painted with Fluon™ to prevent escape.

#### Phorid Flies

**Fig. 2:**
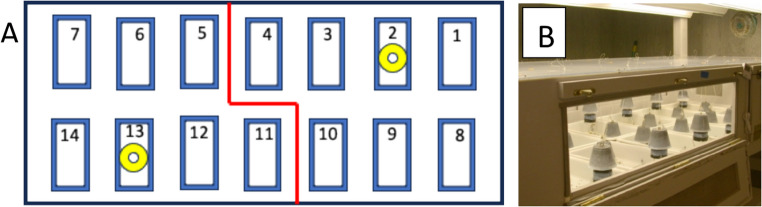
A Phorid fly rearing box schematic used to mass rear phorid flies at DPI. Each rectangle is a tray with fire ant workers and brood. The two trays with yellow donuts received either the bioassay control or treatment. The trays were fitted with two upside down cups that periodically alternated up and down positions, continuously exposing worker ants to attacking phorid flies (Fig. 2B). Photo by Jeffery W. Lotz - Florida Department of Agriculture and Consumer Services, Division of Plant Industry, Gainesville, FL

The *Pseudacteon tricuspis* flies and rearing attack boxes used in this study were produced and maintained at the biocontrol rearing facilities of the Florida Department of Plant Industry (DPI) in Gainesville FL. This facility was engaged in mass rearing the flies for distribution throughout the fire ant infested areas in the southern USA. The phorid flies were reared in automated attack boxes like those described in Vogt et al. (2003) for rearing *P. curvatus*. Each attack box contained two longitudinal rows of seven trays (Fig. 2A), each containing 300 to 400 ants and 1.2 g of brood. The inner sides of the trays were painted with Fluon™ to prevent ant escape. Each attack box tray contained an apparatus consisting of two upside down cups suspended from strings attached to a mechanism that, in seesaw fashion, raised one cup and lowered the other cup. The worker ants instinctively moved their brood under the lowered cup, but after 10 min the two cups changed positions – the up cup moved to the bottom of the tray exposing the previously covered ants and brood (Fig. 2B). Thus, the worker ants were continuously exposed to phorid fly attacks. The large number of phorid fly rearing boxes available eliminated potential pseudo replication issues. The room in which the attack boxes were kept was maintained at 27 ± 2 °C, 60% relative humidity, and a 12:12 L: D photoperiod.

The bioassay utilized tray 2 and 13 for the placement of controls or treatments. Prior to a bioassay, pre-counts of attacking flies were made in the two rearing box sections (Fig. 2A), one for the control and the other for the treatment. If the number of attacking flies was skewed on one side or the other, or if any other abnormality was observed the experiment was terminated, and another rearing box was selected for evaluation. Rearing boxes were not used in bioassays more than once in 24 h. Counting the attacking flies required 2 or more experienced support staff, since the evaluation times were at 2, 5 and 10 min after introduction of the test material.

#### Dufour’s Glands (Recruitment Pheromone)

Fifty Dufour’s glands were dissected as in Vander Meer et al. (2002), and each was placed in the same 500 µL of hexane. For each phorid fly evaluation, a 125 µL aliquot (12.5 worker equivalents) of extract was removed and concentrated under a slow stream of nitrogen to 30 µL (the sample was not allowed to go to dryness). A pre-count of attacking phorid flies was recorded, then the 30 µL of treatment was applied to a 3.5 cm diameter Whatman #1 filter paper circle, and the solvent passively evaporated (ca. 30 s). At the same time 30 µL of hexane (control) was applied to an identical filter paper circle, and the solvent passively evaporated. The treatment and control filter paper circles were immediately put into Petri dishes (6 cm dia.) and placed in the appropriate tray in the attack box. Attacking phorid flies were recorded at 2, 5, and 10 min after the treatment and controls were placed in the attack box. The experiment was replicated 4 times.

#### Venom Sac Contents (Alkaloids+)

Fifty venom sacs (gland and reservoir) were dissected as in Vander Meer et al. (2002), and each was extracted in the same 500 µL of hexane. For each phorid fly evaluation, a 125 µL aliquot (12.5 worker equivalents) of extract was removed and concentrated under a slow stream of nitrogen to 30 µL (the sample was not allowed to go to dryness). A pre-count of attacking phorid flies was recorded, then the entire 30 µL of test material was applied to a 3.5 cm diameter Whatman #1 filter paper circle, and the solvent passively evaporated (ca. 30 s). At the same time 30 µL of hexane was applied to an identical filter paper circle, and the solvent passively evaporated. The treatment and control filter paper circles were immediately put into Petri dishes (6 cm dia.) and placed in the appropriate tray in the attack box. Attacking phorid flies were recorded at 2, 5, and 10 min after the treatment and control were placed in the attack box. The experiment was replicated 4 times.

#### Purified Venom Alkaloids

Fire ant venom alkaloids were isolated from 1.4 kg of *S. invicta* workers (~ 1.4 million workers or about 7 mature colonies). The ants were extracted in hexane. The extract was vacuum filtered through a Buchner funnel, and the collected hexane was vacuum evaporated and then carefully applied to a large gravity silica gel column (see Vander Meer et al. 1988). The column was eluted sequentially with hexane, chloroform, and methanol. The progress of the elution process was followed by spotting each fraction on thin layer chromatography plates and detection of organic compounds with a Phosphopolymolibdic acid (PMA) spray (5% in ethanol) followed by careful heating with an electric heat gun. For example, when compounds stopped eluting via PMA detection from each eluting solvent the solvent was changed. Gas chromatography (GC) demonstrated that the methanol fraction contained large amounts of fire ant venom alkaloids. The venom alkaloids were isolated by removing the methanol by vacuum evaporation, then hexane (400 mL) was added to the residue and shaken to form a solution. The hexane solution was washed 3 times with 200 mL of 2 N HCl/Methanol (1:1) in a separatory funnel and the separated aqueous layers were combined. The acidic aqueous solution, containing alkaloid HCl salts, was made basic using 10% NaOH (converting the HCl salts to free alkaloids). The resulting mixture was extracted 3 times with 200 mL chloroform in a separatory funnel. The chloroform washes were combined and dried with anhydrous sodium sulfate. The combined dry chloroform extracts were analyzed by GC-mass spectroscopy and found to be composed of the expected fire ant piperidine venom alkaloids. The above process was repeated two more times and after GC analysis showed the same result as in the first extract, the three chloroform extracts were combined and evaporated to constant weight yielding *S. invicta* venom alkaloids (ca. 4 g). Venom alkaloid samples were prepared for evaluation of their effect on phorid flies by making a methanol venom alkaloid solution at a concentration of about 3 worker equivalents (30 µg/20 µL methanol). Immediately after pre-counts of attacking phorid flies were made, 20 µL of the alkaloid solution was applied by syringe to a filter paper circle (3.5 cm dia.) in a 6 cm Petri dish. Using a different syringe, 20 µL of methanol was applied to another filter paper circle in a second Petri dish. Both the alkaloid treatment and methanol control were allowed to dry, then both Petri dishes were placed appropriately in the attack box trays. Attacking phorid fly counts were made at 2, 5 and 10 min after introductions.

#### Fire Ant Alarm Pheromone, Shaken Workers

Worker fire ants were previously shown to release alarm pheromones (Vander Meer et al. 2002). Approximately 25 workers ants were placed in each of two 20 mL scintillation vials. Just prior to introduction into an attack box bioassay one of the two vials was shaken. Immediately thereafter, the lids were removed, and the vials were appropriately placed in control and treatment attack box trays. Attacking phorid fly counts were made 1 min after introductions. The experiment was repeated 10 times using different worker ants for each replicate. The first experiment was repeated except the control and shaken ant vials were left open for 4 min prior to introduction into the attack boxes. Attacking phorid fly counts were made 1 min after introductions. The experiment was repeated 10 times using different worker ants for each replicate.

#### Synthetic Pyrazine Alarm Pheromone

The fire ant alarm pheromone was identified as 2-ethyl-3,5-dimethylpyrazine (Vander Meer et al. 2010). It is commercially available as a mixture with the 2-ethyl-3,6-dimethylpyrazine isomer (Aldrich Chemical Co, Milwaukee, WI, USA). The synthetic pyrazine mixture was dissolved in mineral oil at 5ug pyrazine/10uL light mineral oil. The mineral oil slows the release rate of the highly volatile pyrazines.

#### Statistics

All statistical analyses and graphical representations were performed using GraphPad Prism 10.1.2. The controls for the field evaluation of phorid fly attraction to electrically stimulated fire ant workers resulted in zero observed phorid flies, necessitating the use of the Wilcoxon signed rank (non-parametric) test for statistical analysis. For bioassay results in phorid fly rearing boxes the control and treatment data were considered paired and were analyzed using two-tailed t-tests to compare the mean bioassay responses. All quantities are reported as mean ± standard error (SE).

## Results

**Fig. 3:**
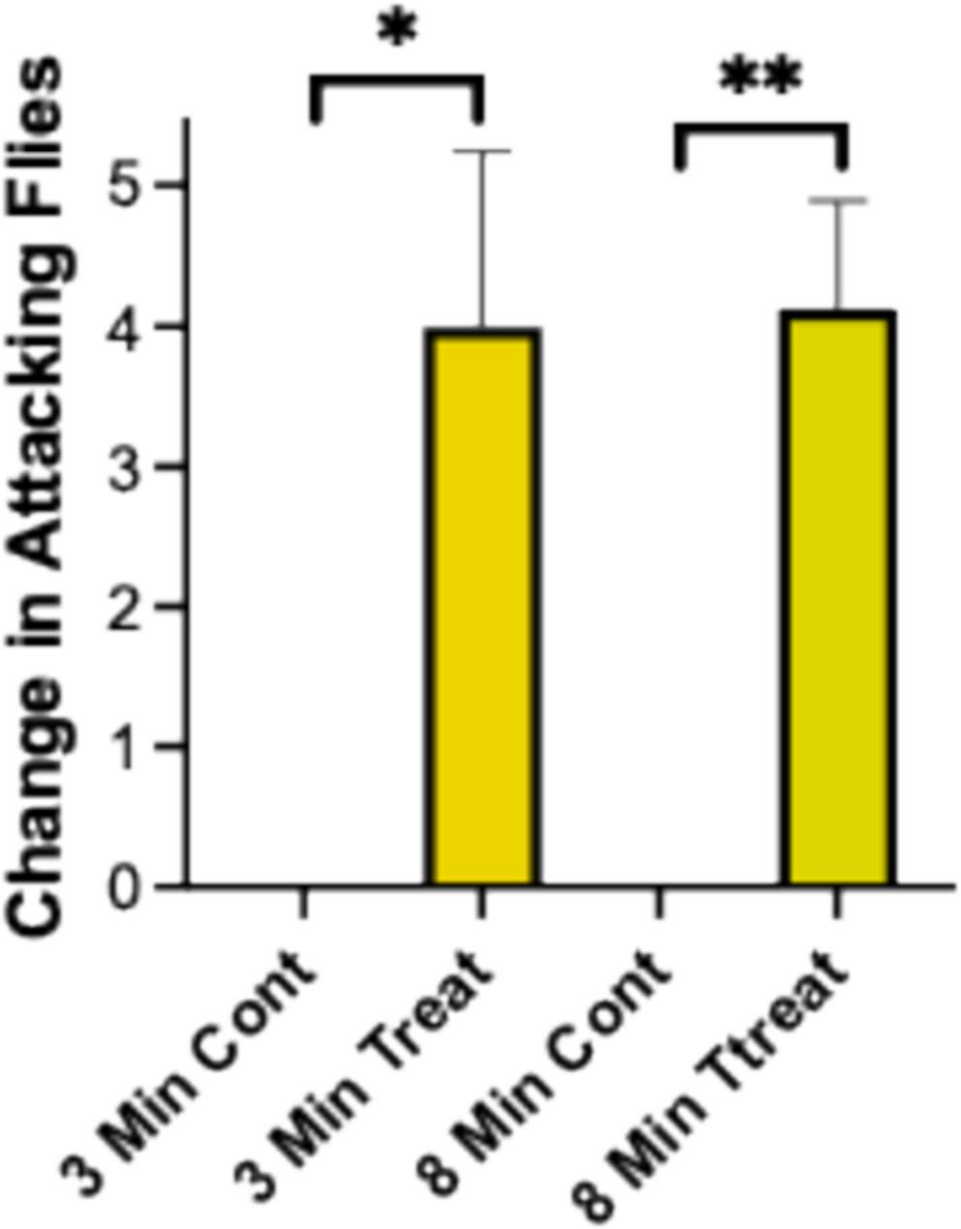
Field Evaluation of Phorid Fly Attraction to Electrically Stimulated Fire Ant Workers. The mean increase in attacking phorid flies is shown for controls and treatments at 3 and 8-min post electrical stimulation (Mean ± SE, * = *P* < 0.01; ** = *P* < 0.001)

No phorid flies were observed at or near fire ant colonies within the evaluation area of the field site. Care was taken to not disturb the colonies, which is known to attract phorid flies (Morrison and King 2004). In most cases, phorid flies were observed near the treatment Petri dish within two minutes after the start of the three-minute electrical stimulation cycle. No flies were attracted to the control Petri dishes containing quiescent worker ants. The number of phorid flies attracted to the electrically stimulated ants and controls are shown in Fig. 3. The treatment attracted significantly greater numbers of flies than the non-electrically stimulated ants in the controls at 3 min after electrical stimulation (Wilcoxon signed rank test, *t* = 3.191, *df* = 7, *P* = 0.0153, *N* = 8), and 8 min after electrical stimulation was stopped (Wilcoxon signed rank test, *t* = 5.384, *df* = 7, *P* = 0.0010, *N* = 8).

### The Effect of Fire Ant Poison Sac Extracts, and Purified Venom Alkaloids on Phorid Fly Activation

**Fig. 4:**
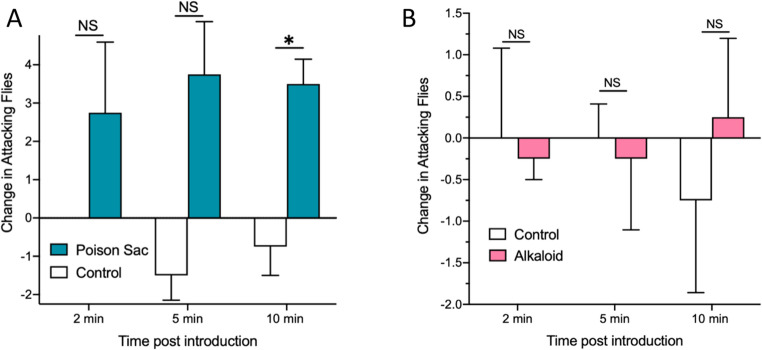
**A**) The means with SE (*N* = 4) are shown for the change in the number of attacking phorid flies in attack box trays containing poison sac extract treatments and solvent controls at 2, 5, and 10 min after introduction. Only the 10 min evaluation time was significantly different from the control (* ; *P* < 0.03, NS = Not significantly different). **B**) The means + or - SE (*N* = 4) are shown for the change in the number of attacking phorid flies in attack box trays containing purified venom alkaloids. NS = not significantly different

Electrical stimulation of workers results in the release of venom products, primarily large amounts of venom alkaloids (Vander Meer et al. 2002). The response of phorid flies in attack boxes to extracts of dissected fire ant worker poison sacs is shown in Fig. 4A. The difference in the number of attacking phorid flies in attack box trays containing poison sac extract treatments and solvent controls at 2 and 5 min after introduction were not significantly different in paired two-tailed t-tests (t = 1.492, df = 3, P = 0.2324; t = 1.492, df = 3, P = 0.2324, respectively). However, the number of attacking phorid flies in response to poison sac extracts at 10 min was significantly different (t = 14.123, df = 3, P = 0.0259). In a separate experiment purified venom alkaloids from a large-scale worker extract were similarly evaluated for their effect on phorid flies (Fig. 4B). Purified alkaloids and their solvent controls were compared at 2, 5, and 10 min after introduction into attack boxes. Treatment phorid fly numbers were not significantly different from controls at any time using paired two-tailed t-tests (2 min: t = 0.2928, df = 3, P = 0.7888; 5 min: t = 0.3333, df = 3, P = 0.7608; and 10 min: t = 0.9258, df = 3, P = 0.4228).

### The Effect of Fire Ant Recruitment Pheromones on Phorid Fly Activation (Dufour’s Gland) Extract

**Fig. 5:**
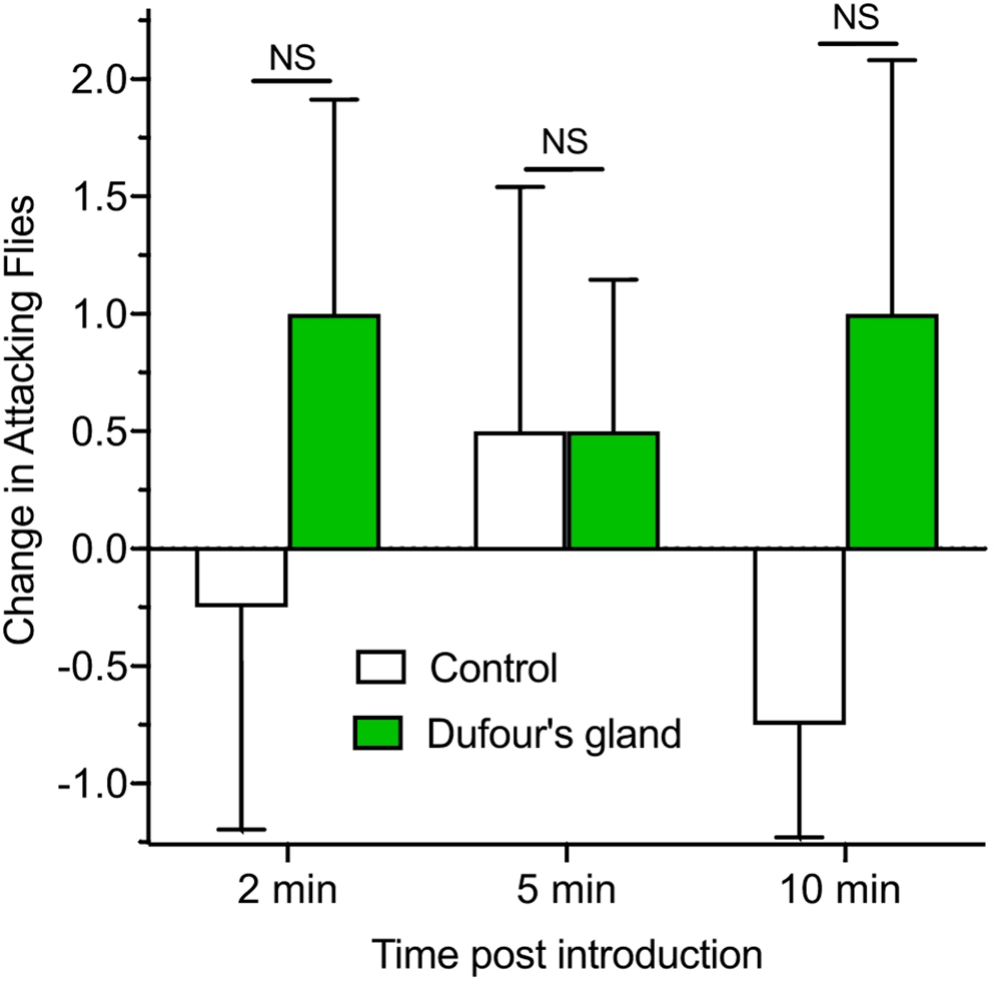
The effect of Dufour’s glands at 12.5 worker equivalents on the number of attacking phorid flies at 2, 5–10 min after introduction were not significantly (NS) different from controls (*N* = 4)

These compounds are multifunctional. If a food source is too large for a foraging worker to bring back to the colony it will lay a chemical trail back to the nest and recruit additional workers to follow the trail back to the large food source. The chemical trail is reinforced by the additional workers, but as the food source diminishes trail reinforcement decreases until food and trail disappear (Wilson 1962). Fire ant recruitment pheromones did not significantly activate *P. tricuspis* phorid flies in rearing box bioassays (Fig. 5) after 2, 5, and/or 10 min post Dufour’s gland extract introduction (*two-tailed t-tests*, *N* = 4; 2 min: P = 0.5367, *t* = 0.6956, *df* = 3; 5 min: *P* = 0.3441, *t* = 1.121, *df* = 3,; and 10 min: *P* = 0.3101, *t* = 1.219, *df* = 3, *N* = 4. NS = Not Significantly different.

### The Effect of Fire Ant Alarm Pheromone on Phorid Fly Activation (Shaken Worker Ants and Synthetic Pyrazine Alarm Pheromone)

**Fig. 6:**
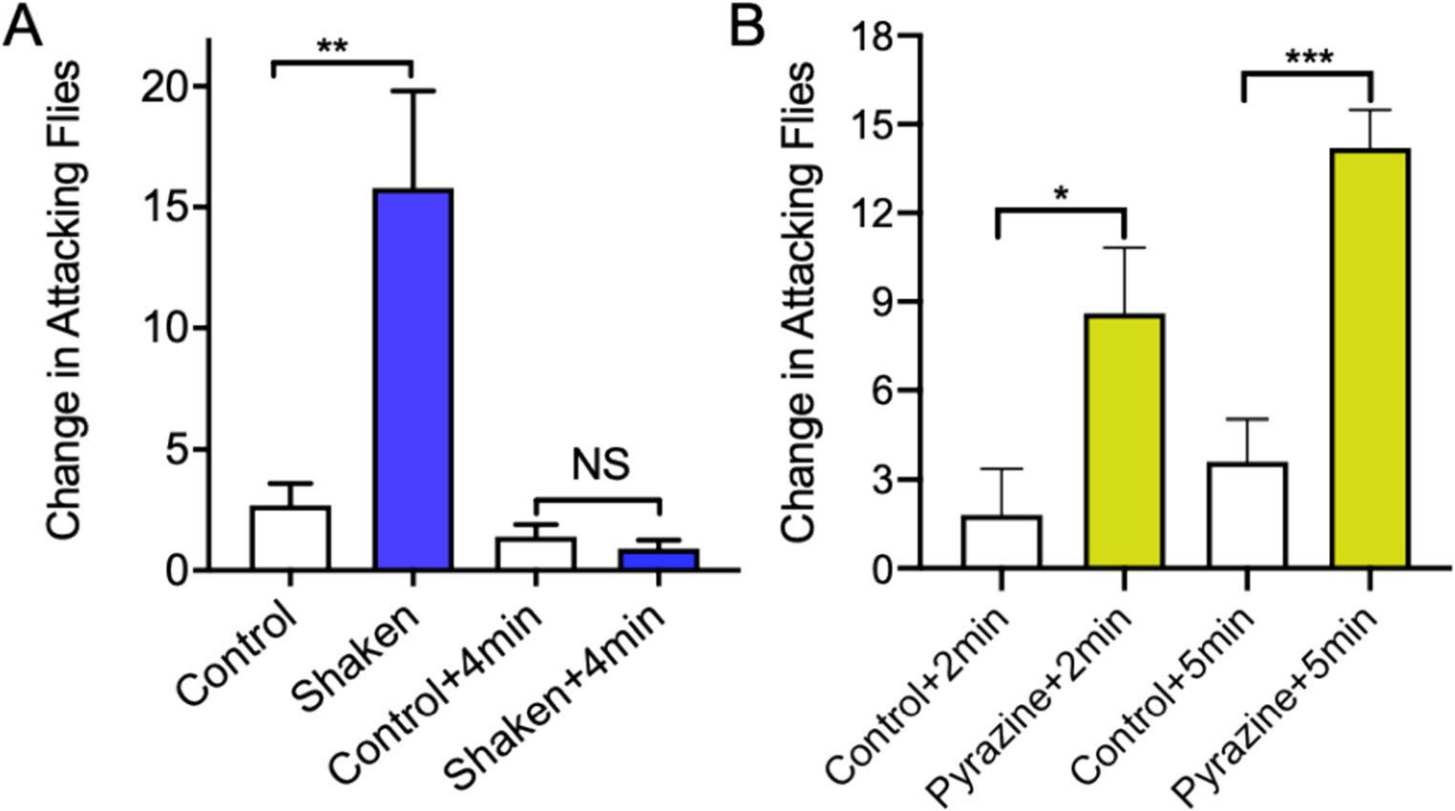
Shaken fire ants release ephemeral alarm pheromones. Figure 6A shows the response of phorid flies in attack boxes to volatiles emanating from vials containing quiescent fire ant workers (control) or agitated (alarmed) workers in shaken vials opened and immediately tested, compared with shaken vials after being open for 4 min before testing. Figure 6B shows the results of placing a mineral oil solution of the synthetic pyrazine alarm pheromone (5ug pyrazine/10uL mineral oil) in the treatment vials. After both 2 and 5 min there were significant increases in attacking phorid flies. NS = not significantly different; * *P* = 0.03; ** *P* = 0.01; *** *P* = 0.001)

The fire ant pyrazine alarm pheromone and an isomer is available (Aldrich Chemical Co., St. Louis, MO). Both isomers were shown to attract phorid flies. Shaken ants were also shown to attract phorid flies. Fire ant alarm pheromones from shaken ants activated *P. tricuspis* flies in rearing box bioassays (Fig. 6A) within the first minute after the shaken ant vial was opened and placed in the appropriate treatment Tray (*P* = 0.0078, 2-tailed, *t* = 0.3.407, *df* = 9, *N* = 10). When the shaken ant vials were kept open for 4 min and then placed in the rearing box bioassay the flies were not activated (*P* = 0.4642, 2-tailed, *t* = 0.7645, *df* = 9, *N* = 10). However, when synthetic pyrazine alarm pheromone in mineral oil was used in the bioassay, phorid flies were significantly activated at both 2 min and 5 min after introduction (*P* = 0.0288, 2-tailed, *t* = 3.342, *df* = 4, *N* = 5; *P* = 0.0009, 2-tailed, *t* = 8.773, *df* = 4, N = 5, respectively).

## Discussion

A previous study demonstrated that fire ants are attracted to exocrine products released by electrically stimulated fire ants, but not the electrical field alone (Vander Meer et al. 2002). This study demonstrates that electrically stimulated fire ants attracted phorids flies under field conditions (Fig. 3), which led to development of systematic methods to isolate glandular sources of fire ant semiochemicals for use in behavioral fire ant/phorid fly bioassays.

Research to determine the source of phorid fly host attractants has been ongoing for the past two decades. Chen and Fadamiro (2007) showed that fire ant whole body, head, and abdomen extracts elicited strong male and female *P. tricuspis* electroantennogram (EAGs) responses. Fire ant thorax extracts elicited a smaller response and E, E-α-farnesene, a minor component of *S. invicta*’s recruitment pheromone produced in the Dufour’s gland and released through the sting (Vander Meer et al. 1988), had no effect on *P. tricuspis* antennae. This farnesene isomer is the only recruitment pheromone component of four identified that is commercially available. EAG responses are interesting, but they are not necessarily informative regarding function. That is why behavioral Y-Tube olfactometer bioassays (Chen and Fadamiro 2007) are more interesting. They showed significant *P. tricuspis* attraction to live *S. invicta* workers, worker whole body and worker thorax extracts, but not to head or abdomen extracts, or the trail pheromone component, E, E-α-farnesene. The authors suggested that the fire ant worker thorax was the source of *P. tricuspis* attractants. While these data do not rule out potential involvement of the fire ant recruitment pheromone in *P. tricuspis* host attraction, our Dufour’s gland results reported here (Fig. 5) clearly demonstrate that the fire ant recruitment pheromone does not attract *P. tricuspis*. This was followed by a report (Chen et al. 2009) that fire ant venom alkaloids (cis- and trans- alkaloid fractions) elicited phorid fly attraction and strong EAG responses from *P. tricuspis* antennae.

The *S. invicta* alarm pheromone was isolated and identified as 2,6-dimethyl-3-ethylpyrazine (Vander Meer et al. 2010). Mandibular glands are the source of this volatile pyrazine alarm pheromone. High volatility and worker sensitivity along with low mandibular gland concentrations contributed to the decades needed to isolate and identify the pheromone. A mixture of the fire ant alarm pheromone and an isomer is available commercially. This mixture was used to demonstrate significant phorid fly EAG responses at concentrations lower than those generated by other pyrazine analogs, in support of a role for the fire ant alarm pheromone in phorid fly attraction to its fire ant host (Sharma et al. 2011). However, since fire ant alkaloids were previously shown by Chen et al. (2009) to attract phorid flies, Sharma et al. (2011) proposed that the two chemical classes may act in tandem. This theme was continued in Sharma and Fadamiro (2013) where they proposed that *P. tricuspis* uses the fire ant alarm pheromone as a long-range attractant and the venom alkaloids as short range attractants. This was reiterated in a comprehensive review of phorid fly host specificity and impact (Chen and Fadimiro 2018).

Our results do not support the Sharma and Fadamiro (2013) proposal that venom alkaloids are short range attractants, since purified alkaloids had no effect on phorid flies when placed in what would be considered a short-range experiment (Fig. 4B). The discrepancy in the two alkaloid results is likely due to the alkaloid isolation method used in the two situations: Chen et al. (2009) used a method they developed to analyze fire ant samples for piperidine alkaloids, e.g., about 5 g of worker ants were soaked in hexane for 24 h, then an extract aliquot was applied to a Pasture pipette (a separation column) containing silica gel/hexane. The column was eluted with increasing ratios of acetone to hexane. Three fractions were collected: #1 was hydrocarbons; #2 was called the cis-alkaloid fraction, and #3 was the trans-alkaloid fraction. Both alkaloid fractions were shown to attract phorid flies (Chen et al. 2009. In contrast, we chemically removed the alkaloids from a worker ant hexane extract by acidifying and isolating alkaloid HCl salts in the aqueous layer, leaving neutral and acidic compounds, including those responsible for the positive phorid fly attraction (Fig. 4A) behind in the hexane extract. It is likely that the cis- and trans- piperidine fractions in Chen et al. (2009) contained other undetected compounds that are responsible for phorid fly attraction. Related to this problem, Shi et al. (2015) reported that the alkaloid profile of *S. geminata* workers collected from Florida, USA contained only cis- and trans- piperidine alkaloids. However, Vander Meer et al. (2022) reported that their procedure masked the presence of several significant pyridine alkaloids. This supports our contention that in Chen et al. 2009; cis- and trans- piperidine fractions likely contained other compounds responsible for phorid fly attraction.

Our results show that venom sac extracts at all 3 time periods had small increases in attacking flies, but only at 10 min was the increase significantly different from the control (Fig. 4A). Interestingly, purified alkaloids resulted in no phorid fly attraction during the experimental timeframe (Fig. 4B). Therefore, we must conclude that while venom alkaloids may elicit an EAD response (Chen et al. 2009), they are not involved in *P. tricuspis* attraction to host fire ants. However, non-alkaloid components produced by the poison gland, may influence phorid fly attraction to or recognition of its fire ant host and warrants further investigation. Venom is used in many ways - defense, prey procurement, nest hygiene, and the queen deposits venom on each egg as it is laid. Future experiments could be carried out using phorid fly rearing boxes as a bioassay tool to guide the chemical separation of poison sac extracts to isolate the compound(s) potentially responsible for low-level phorid fly attraction (Fig. 4A).

Results with synthetic alarm pheromone clearly demonstrate its ability to activate phorid flies to an extent greater than any other exocrine gland extracts evaluated in this study. Alarm pheromones are released with any mound/colony disturbance, e.g., farm animals or farm equipment, during territorial colony to colony conflicts, and during mating flight initiation **(**Morrison and King 2004).

In summary, a series of bioassays demonstrated that electrically stimulated ants are much more attractive to decapitating flies than unstimulated ants. We also found that physically shaken ants are highly attractive to the flies and that this attractiveness disappears rapidly over time, suggestive of the ephemeral nature of the fire ant alarm pheromone. We determined that extracts of the poison sac and the Dufour’s gland were not attractive to the flies. The information provided by these bioassays clearly showed that mandibular gland alarm pheromones were the primary chemical cues *P. tricuspis* flies use to locate their hosts. The results of this study also help explain why *Pseudacteon* decapitating flies are highly host specific (Porter and Gilbert 2004). It is likely that improved knowledge of the mechanisms associated with host attraction can be used to improve field collection, host specificity testing, and mass rearing of *Pseudacteon* flies for release as fire ant biocontrol agents.

## Data Availability

Data generated during research for this paper will be made available upon request.
